# Training Spiking Neural Models Using Artificial Bee Colony

**DOI:** 10.1155/2015/947098

**Published:** 2015-02-01

**Authors:** Roberto A. Vazquez, Beatriz A. Garro

**Affiliations:** ^1^Intelligent Systems Group, Faculty of Engineering, La Salle University, Benjamín Franklin 47, Colonia Condesa, 06140 Mexico City, DF, Mexico; ^2^Instituto en Investigaciones en Matemáticas Aplicadas y en Sistemas, Universidad Nacional Autónoma de México, Ciudad Universitaria, 04510 Mexico City, DF, Mexico

## Abstract

Spiking neurons are models designed to simulate, in a realistic manner, the behavior of biological neurons. Recently, it has been proven that this type of neurons can be applied to solve pattern recognition problems with great efficiency. However, the lack of learning strategies for training these models do not allow to use them in several pattern recognition problems. On the other hand, several bioinspired algorithms have been proposed in the last years for solving a broad range of optimization problems, including those related to the field of artificial neural networks (ANNs). Artificial bee colony (ABC) is a novel algorithm based on the behavior of bees in the task of exploring their environment to find a food source. In this paper, we describe how the ABC algorithm can be used as a learning strategy to train a spiking neuron aiming to solve pattern recognition problems. Finally, the proposed approach is tested on several pattern recognition problems. It is important to remark that to realize the powerfulness of this type of model only one neuron will be used. In addition, we analyze how the performance of these models is improved using this kind of learning strategy.

## 1. Introduction

Artificial neural networks (ANNs) are applied in a broad range of problems. Among the most popular tasks using ANN, we could mention pattern recognition, forecasting, and regression problems. However, the accuracy of these models could drastically diminish if the topology is not well-designed and if the training algorithm is not selected carefully. One interesting alternative to designing the topology and training and exploiting the capabilities of an ANN is to adopt a learning strategy based on evolutionary and swarm intelligence algorithms. It is well-known that designing and training tasks can be stated as optimization problems; for that reason, it is possible to apply different types of evolutionary and swarm intelligence algorithms. For example, particle swarm optimization [1] and differential evolution [2] have been used to design and train ANNs automatically.

Several swarm intelligence algorithms based on the collective behavior of self-organizing systems have been proposed in the last years. Among the most popular, we could mention ant colony system (ACO) [3] and particle swarm optimization (PSO) [1] and artificial bee colony (ABC) [4]. Most of the studies related to honey bee swarm are focused on the dance and communication, task allocation, collective decision, nest site selection, mating, marriage, reproduction, foraging, floral and pheromone laying, and navigation behaviours of the swarm [5]. ABC is a novel algorithm that tries to mimic the behavior of the bees in nature, which tasks consist in exploring their environment to find a food source.

The ABC algorithm has been used in a broad range of optimization problems. This algorithm is relatively simple, and its implementation is straightforward for solving optimization problems, being able to produce acceptable results at a low computational cost. The different studies performed in the literature compare its efficiency against other traditional strategies such as genetic algorithm (GA), differential evolution (DE), particle swarm optimization (PSO), ant colony optimization (ACO), and their variants. The efficiency obtained on numerical problems using numerical test functions, multivariable functions, constrained and unconstrained optimization problems, and even multiobjective problems, suggests that the ABC algorithm is a serious candidate for training ANN [5].

Some of the first works that use the ABC algorithm to adjust the synaptic weight of an ANN are described in [6, 7]. In [8] the authors train a feed-forward ANN using ABC applied to solve the XOR, 3-bit parity, and 4-bit encoder-decoder problem, and some signal processing applications. In [9], the ANN is trained using ABC algorithm to solve a medical pattern classification problem. In [10], the authors train an ANN to classify different dataset utilized in the machine learning community. In [11], the ANN is trained for the classification of the acoustic emission signal to their respective source. Another interesting paper for designing and training an ANN is presented in [12], where the authors described a methodology for maximizing its accuracy and minimizing its connections by evolving the weights, the architecture, and the transfer function of each neuron. In the context of forecasting, [13] used ABC to train an ANN for bottom hole pressure prediction in underbalanced drilling. Whereas in [14] the authors train a recurrent ANN for stock price forecasting, in [15] the author uses ABC for training an ANN for earthquake time series data. Reference [16] presents an ANN trained with ABC for S-models of biomedical networks approximation. From all these papers the authors conclude that ABC algorithm is capable of training ANN with an acceptable accuracy and, in some cases, the results are better than those obtained with other traditional techniques.

Moreover, there exist many algorithms based on the bees' behavior such as bee algorithm, honey-bee mating algorithm, and bee colony optimization (BCO), among others [17]. Hence, there are investigations about training ANNs that use different kinds of algorithms related to the ABC. For example, in [18] the authors apply the bee colony algorithm to train an ANN, which later is applied to the wood's defect problem. In [19], the authors estimate the state variables in distribution networks, including distributed generators using a honey-bee mating algorithm. Furthermore, in [20], the authors use this algorithm in combination with a self-organizing map (SOM) in the market segmentation problem.

Swarm intelligence algorithms have contributed and gained popularity in the field of ANN as a learning strategy. However, the intrinsic limitations of ANN do not allow applying them in complex pattern recognition problems, even using different learning strategies. These limitations motivate to explore other alternatives to model and generate neural models to make possible their application in several pattern recognition problems.

Although ANNs were inspired by the behavior of the human brain, the fact is that they do not mimic the behavior of a biological neuron. In that sense, the development and application of more realistic neural models could improve the accuracy of an ANN during several pattern recognition tasks.

Spiking neuron models are called the 3rd generation of artificial neural networks [21]. These neurons increase the level of realism in a neural simulation and integrate the concept of time. These types of models have been used in a broad range of areas, mainly from the field of computational neurosciences [22], brain region modeling [23], auditory processing [24, 25], visual processing [26–28], robotics [29, 30], and so on.

Several spiking models have been proposed in the last years. One of the most realistic and complex models was proposed in [31]. Nonetheless, there are simplified versions of this model that reduce its computational complexity. Among these models, we could mention the well-known integrate-and-fire model [32], Izhikevich model [33], FitzHugh-Nagumo model [34], and Hindmarsh-Rose [35].

Theoretically, these types of models could simulate the behavior of any perceptron type neural network. However, their application in computer vision and pattern recognition has not been widely explored. Although there are some works related to image segmentation [36–38] and pattern recognition [39–43], there still are several issues to research related to the learning process, design, and implementation.

The process of learning of these models is conducted with different techniques. In [44], the authors present the Spike-Prop, an adaptation of the well-known backpropagation algorithm to train a spiking neural model. Furthermore, several variants to improve the efficiency of spike-prop have been proposed [45–47]. However, these algorithms require a careful tuning of the network to obtain acceptable results.

Another approach to train these models is based on probabilistic models [47–49], information bottleneck learning [50–52], and reinforcement learning [53–55].

On the other hand, nongradient based methods like evolutionary strategies (such as GA, PSO, and DE) have emerged as an alternative to traditional methods for training spiking neural models. Although this approach is computationally more expensive compared with traditional methods, it has several advantages that make possible its application in real pattern recognition problems [42, 56, 57].

Recently, it has been proven that only one spiking neuron model can solve nonlinear pattern recognition problems, showing a clear advantage against the traditional perceptron [58–60]. One alternative to simulate the learning process of this type of model is to use swarm intelligence algorithms. For example, in [58] the authors describe an approach to applying a leaky-integrate-and-fire spiking neuron in various linear and nonlinear pattern recognition problems. In that work, the authors use the differential evolution algorithm as a learning strategy. In other researches, the authors use the Izhikevich spiking model to the same set of problems using a differential evolution strategy [60]. In [61, 62], the authors use the Izhikevich spiking model to the same set of problem using cuckoo search and particle swarm optimization algorithms, respectively. In general, the methodology described in those papers can be stated as follows: given a set of input patterns belonging to *K* classes, first of all, each input pattern is transformed into an input signal. Then the spiking neuron is stimulated during *T* ms and the firing rate is computed. After adjusting the synaptic weights of the spiking model by means of a swarm intelligence algorithm, we expect that input patterns from the same class produce similar firing rates, and input patterns from different classes generate firing rates different enough to discriminate among the categories.

Despite the results presented in those papers, it is still necessary to explore and develop strategies that allow these models to learn from their environment and improve their accuracy solving complex pattern recognition problems. Due to the capabilities of producing acceptable results at a low computational cost in a broad range of optimization problems, including ANN field, the ABC algorithm could be an excellent tool for simulating the learning process of a spiking neural model.

In this paper, we proposed to use the ABC algorithm as a learning strategy to training a spiking neuron model aiming to perform various linear and nonlinear pattern recognition problems. Based on the methodology described in [60, 61], we present a comparison of the results presented in [58, 60, 61] to determine which learning strategy provides the best accuracy and how it affect the accuracy of the spiking neuron. In order to test the accuracy of the learning strategy combined with the spiking neuron model, we perform several experiments using different pattern recognition problems, including an odor recognition problem and cancer classification based on DNA microarrays.

The outline of this paper is divided into five sections. A brief introduction to the ABC algorithm is presented in Section 2. The concepts related to the third generation of neural networks knowing as spiking neural networks are presented in Section 3.  Section 4 presents the proposed methodology for training spiking neural networks using the ABC algorithm. The experimental results, as well as the discussion of the results, are presented in Section 5. Finally, the conclusions of this work are presented in Section 6.

## 2. Basics on Artificial Bee Colony

The artificial bee colony (ABC) algorithm is a novel approach in the area of the swarm optimization proposed by Karaboga and Akay [6]. The ABC algorithm is based on the behavior of bees in nature, whose task consists of exploring their environment to find a food source, picking up the flower's nectar and returning to the hive in order to evaluate the quality and the amount of the food, and then call the other bees of the community to fly towards the food source. Communication among bees is done by a particular dance.

This algorithm can find the optimum values in the search space of a given optimization problem. A global optimization problem can be defined as finding the parameter vector **x** that minimizes the objective function *f*(**x**):

(1) $$\begin{array}{c} \text{minimize}\;f\left(x\right),\;x=\left(x_{1},x_{2},\ldots ,x_{i},\ldots ,x_{n-1},x_{n}\right)\in R^{n} \end{array}$$

which is constrained by the following inequalities and/or equalities:

(2) $$\begin{array}{c} l_{i}\leq x_{i}\leq u_{i},\;i=1,\ldots ,n. \end{array}$$

*f*(**x**) is defined on a search space, *S*, which is *n* dimensional rectangle in *R* ^*n*^. The variable domains are limited by their lower and upper bounds (2).

This algorithm represents the solutions of a given problem by means of the position of different food sources visited by a bee. Furthermore, it works with three kinds of bees in order to explore and exploit the search space: employed, onlookers, and scouts bees.

Following the work described in [7], the employed bee has to modify the position (solution) in its memory based on the local information (visual information) and test the nectar amount (fitness value) of the new source (new solution). If the quantity of nectar in the new position is better than the old one, the bee memorizes it and forgets the old one. Contrarily, it keeps the previous one in its memory. After the entire employed bees complete the search process, they share the nectar information about the food sources and their position, with the onlooker bees in the dance area.

An onlooker bee checks the nectar information obtained from all employed bees and selects a food source with a probability in terms of its nectar amount. The employed bee modifies the position in its memory and checks the quantity of nectar obtained from the candidate source. If its nectar is higher than that of the previous one, then the bee memorizes the new position and forgets the old one.

An artificial onlooker bee chooses a food source depending on the probability *p*_*i*_ associated with that food source. This probability is calculated with the following expression:

(3) $$\begin{array}{c} p_{i}=\frac{{\text{fit}}_{i}}{\sum_{n=1}^{\text{SN}}{\text{fit}}_{n}}, \end{array}$$

where fit_*i*_ is the fitness value of the solution *i* of dimension *D* which is proportional to the nectar amount of the food source in the position *i* and SN is the number of food sources that is equal to the number of employed bees.

In order to produce a candidate food position from the old one in memory, the ABC algorithm uses the following expression:

(4) $$\begin{array}{c} v_{ij}=x_{ij}+\phi_{ij}\left(x_{ij}-x_{kj}\right), \end{array}$$

where *k* ∈ {1,2,…, SN} and *j* ∈ {1,2,…, *D*} are indexes randomly chosen. Although *k* is determined randomly, it has to be different from *i*. *ϕ*_*ij*_ is a random number between [−1,1] that controls the production of neighbor food sources around *x*_*ij*_.

The food source whose nectar is discarded by the bees is changed with a new food source by the scouts. In the ABC algorithm, this is simulated by producing a random position and replacing it with the abandoned one. If that position cannot be enhanced after a number of trials, then the solution is discarded. This parameter is called the “limit” for abandonment. Assume that the abandoned source is *x*_*i*_ and *j* ∈ {1,2,…, *D*}; then the scout bee discovers a new food source to be replaced with *x*_*i*_. This operation can be defined as

(5) $$\begin{array}{c} x_{ij}=l_{j}+\text{rand}\left(0,1\right)\left(u_{j}-l_{j}\right), \end{array}$$

where *l*_*j*_ and *u*_*j*_ are the lower and upper bounds of the parameter *x*_*ij*_, respectively.

After each candidate source position *v*_*ij*_ is generated and then evaluated by the artificial bee, its performance is compared to the performance associated with the previous position. If the new food source has equal or better nectar than the old one, then it is substituted with a new one in the memory. Otherwise, the old one is retained in the memory.

The pseudocode of the ABC algorithm is shown in Pseudocode 1.

The ABC algorithm randomly initializes a population of solutions that represent the position of food sources within the lower and upper bounds. The size of the population is a parameter that corresponds to the number of food sources that is equal to the number of employed or onlooker bees. The stop criterion adopted in this algorithm is the maximum cycle number. The solutions are limited by their lower and upper bounds (2). If they are out of the boundaries, they are set to the lower or upper bounds.

One advantage of this algorithm is that only three control parameters are needed: population size (SN), the maximum cycle number (MCN), and the value of the “limit.”

## 3. Spiking Neural Models

The main distinctive elements that compose a typical spiking neuron are: dendrites (*input device*), soma (*central processing unit*), and axon (*output device*). The dendrites collect signals from other neurons and transmit them to the soma. The soma performs an important nonlinear processing step where an output signal is generated if the total input exceeds a certain threshold. Finally, the output signal is taken over by the axon, which delivers the signal (short electrical pulses called action potentials or spikes) to other neurons. Typically, the spikes have an amplitude of about 100 mV and a duration of 1-2 ms [63]. Although the same elements exist in a linear perceptron, the main difference between a linear perceptron and a spiking model is the action potential generated during the stimulation time. Furthermore, the activation function used in spiking models is a differential equation that tries to model the dynamic properties of a biological neuron in terms of spikes.

According to [63], a spike train is a sequence of stereotyped events generated at regular or irregular intervals. The form of the spike does not carry any information, and what is important is the number and the timing of spikes. The shortest distance between two spikes defines the absolute refractory period of the neuron that is followed by a phase of relative refractoriness where it is difficult to generate a spike.

Several spiking models have been proposed in the last century aiming to model different neurodynamic properties of neurons [64]. Among these models, we could mention the well-known integrate-and-fire model, resonate-and-fire, Izhikevich model, FitzHugh-Nagumo model, and Hodgkin-Huxley model.

One of the most simple and versatile models is the one proposed by Izhikevich. This model has only nine dimensionless parameters, and it is described with the next equation:

(6) Cv˙=kv−vrv−vt−u+I if  v≥vpeak  thenu˙=abv−vr−u v⟵c,  u⟵u+d.

Depending on the values of *a* and *b*, the spiking model can act as an integrator or a resonator. Whereas *a* is the recovery time constant, the sign of *b* force to *u* behaves as an amplifying (*b* < 0) or a resonant (*b* > 0) variable. The parameters *c* and *d* consider that the action of high-threshold voltage-gated currents activated during the spike affects only the after-spike transient behavior. In addition, *c* define the voltage reset value, and *d* describes the total amount of outward minus inward currents activated during the spike. On the other hand, the variable *v* represents the membrane potential, *u* is the recovery current, *C* is the membrane capacitance, *v*_*r*_ is the resting membrane potential, *v*_*t*_ is the instantaneous threshold potential, and *v*_peak_ is the spike cutoff value [65].

**Figure 1:**
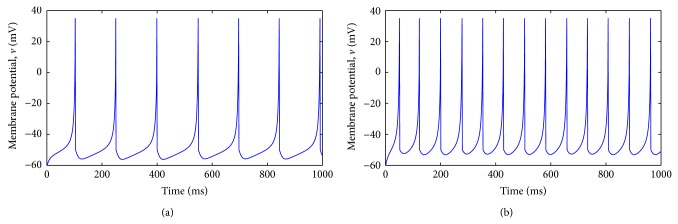
Simulation of the Izhikevich neuron model $100\dot{v}=0.7\left(v+60\right)\left(v+40\right)-u+I$, $\dot{u}=0.03\left\{-2\left(v+60\right)-u\right\}$, if *v* ≥ 35 then *v* ← −50 and *u* ← *u* + 100. (a) Injection of the step of DC current *I* = 70 pA. (b) Injection of the step of DC current *I* = 100 pA.

According to [33], the spiking model can reproduce various intrinsic firing patterns based on different values of the parameters. Regular spiking (RS) neurons are the most typical neurons in the cortex; see Figure 1. For the intrinsically bursting (IB) behavior, the neurons fire a stereotypical burst of spikes followed by repetitive single spikes. Whereas the neurons with a chattering (CH) behavior can fire stereotypical bursts of closely spaced spikes, neurons with a fast-spiking (FS) behavior can fire periodic trains of action potentials with extremely high frequency practically without any adaptation (slowing down). Finally, for the low-threshold spiking (LTS), the neurons can also fire high-frequency spike trains but with a notable spike frequency adaptation.

A profound description of the Izhikevich model can be found in [65]. In this paper, we will concentrate on the parameters that produce regular spiking patterns. However, we do not discard the use in the near future of other firing patterns, useful for solving pattern recognition tasks. The Izhikevich model can produce a class 1 neural excitability behavior: action potentials can be generated with arbitrary low firing rate, depending on the strength of the applied current [65]. The response of the Izhikevich neuron changes if the input current changes generating different firing rates; see Figure 1.

The neuron is stimulated during *T* ms with an input signal, and it fires when its membrane potential reaches a specific value and thus generating an action potential (spike) or a train of spikes.

## 4. Proposed Method

Based on the hypothesis “patterns from the same class produce similar firing rates in the output of the spiking neuron and patterns from other classes produce firing rates different enough to discriminate among the classes,” the authors in [58, 60] proposed a methodology which describes the way that a spiking neuron can be applied to solve pattern recognition problems.

Following the same approach, let *D* = {**x** ^*i*^, *k*}_*i*=1_^*p*^ be a set of associations composed of *p* input patterns, where *k* = 1,…, *K* is the class to which **x** ^*i*^ ∈ *R* ^*n*^ belongs. The learning process adjusts the synaptic values of the spiking model in such way that the output generates a different firing rate for each class *k*, reproducing the behavior described in the hypothesis.

### 4.1. Classifying with Firing Rates

This subsection describes how a spiking neural model can be applied in a pattern classification task based on [58, 60].

In order to use a spiking neural model to solve a pattern classification problem, it is necessary to compute the input current *I* that stimulates the model. In other words, the spiking neuron model is not directly stimulated with the input pattern **x** ^*i*^ ∈ *R* ^*n*^ but with the input current *I*. If we assume that each feature of the input pattern **x** ^*i*^ corresponds to the presynaptic potential of different receptive fields, then we can calculate the input current *I* that stimulates the spiking neuron as

(7) $$\begin{array}{c} I=x\cdot w, \end{array}$$

where **w** ^*i*^ ∈ *R* ^*n*^ is the set of synaptic weights of the neuron model. This input current is used in the methodology to stimulate the spiking model during *T* ms.

Instead of using the spike train generated by the spiking model to perform the pattern classification tasks, we compute the firing rate of the neuron defined as

(8) $$\begin{array}{c} \text{fr}=\frac{N_{\text{sp}}}{T}, \end{array}$$

where *N*_sp_ is the number of spikes that occur within the time window of lenght *T*.

It is necessary to calculate the average firing rate AFR ∈ *R* ^*K*^ of each class, by using the firing rates produced by each input pattern. In this sense, the learning process consists of finding the synaptic values of the spiking model in such way that it generates a different average firing rate for each class *k*.

Suppose that the spiking neuron is already trained using a learning strategy. To determine the class to which an unknown input pattern **x** belongs, it is necessary to compute the firing rate generated by the trained spiking neuron. After that, the firing rate is compared against the average firing rate of each class. The minimum difference between the firing rate and the average firing rates determines the class of an unknown pattern. This is expressed with the following equation:

(9) $$\begin{array}{c} \mathrm{cl}=\arg\overset{K}{\underset{k=1}{\min}}\left(\left|{\text{AFR}}_{k}-\text{fr}\right|\right), \end{array}$$

where fr is the firing rate generated by the neuron model stimulated with the unknown input pattern $\tilde{x}$.

### 4.2. Adjusting Synapses of the Spiking Neuron Model

In order to achieve the desired behavior at the output of the spiking neuron, it is necessary to adjust its synaptic weights. This step corresponds to the learning (training) phase of the spiking neuron. Several swarm intelligence algorithms can be used in the learning phase, but in this research we focus on the artificial bee colony (ABC) algorithm as a learning strategy.

The synapses of the neuron model **w** are adjusted by means of the ABC algorithm. The food source in the ABC algorithm represents the synaptic weights of the spiking model. In order maximize the accuracy of the spiking neuron model during a pattern recognition task, the best set of synaptic weights must be found using the ABC algorithm. However, if we state the problem as a minimization problem, the classification error must be minimized. The fitness function that uses the classification error to find the set of synaptic weights is defined as

(10) $$\begin{array}{c} f\left(w,D\right)=1-\text{performance}\left(w,D\right), \end{array}$$

where **w** are the synapses of the spiking model, *D* is the set of input patterns, and performance(**w**, *D*) is a function which computes the classification accuracy, in terms of (9), given by

(11) $$\begin{array}{c} \text{performance}\left(w,D\right)=\frac{P_{\text{cc}}}{P_{t}}, \end{array}$$

where *P*_cc_ denotes the number of patterns correctly classified and *P*_*t*_ denotes the number of tested patterns.

## 5. Experimental Results

In this section, we analyze and discuss the results generated with the proposed methodology. In order to evaluate the accuracy of the proposed methodology, we perform several experiments using different datasets. The section is divided into three subsections. The first section presents a comparison of the proposed methodology against different swarm intelligence algorithms. In the last two subsections, we present preliminary results applying spiking neural models using the proposed methodology for solving an odor classification problem and cancer classification based on DNA microarrays.

### 5.1. Analysis and Comparison of Experimental Results

In order to evaluate the classification accuracy, when the spiking neuron is trained using the ABC algorithm, six sets of experiments were performed. Each set of experiments was performed with a dataset that corresponds to a specific pattern recognition problem. The iris plant, glass, diabetes, liver-bupa, and wine datasets were taken from the UCI machine learning benchmark repository [66].

The iris plant dataset is a three-class problem composed of input patterns with four features. The wine dataset also is a three-class problem composed input patterns with 13 features. The glass dataset is a six-class problem composed of patterns with nine features; however, in this experiment we used the two most representative classes. The diabetes dataset is a two-class problem composed of input patterns with eight features. Finally, the liver dataset also is a two-class problem composed of input patterns with six features.

**Figure 2:**
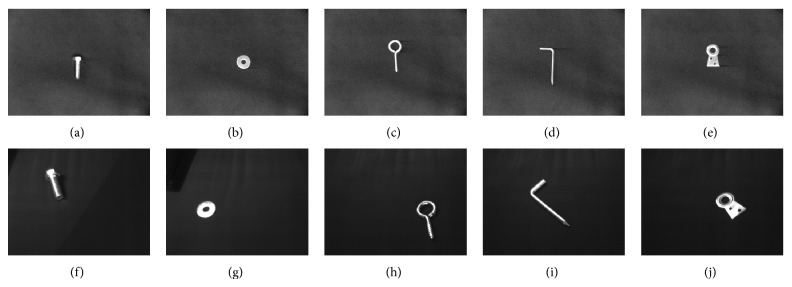
(a)–(d) Some of the images used to train the proposed method. (f)–(i) Some of the images were used to test the proposed method.

Furthermore, an object recognition problem, composed of five different objects, was used to evaluate the accuracy of the proposal; see Figure 2 [67]. A dataset was generated from a set of 100 images in different positions, rotations, and scale changes. Instead of recognizing objects directly from their images, an invariant description of each object was calculated applying the next process over each image: a standard threshold [68] was applied to get its binary version; small spurious regions were eliminated from each image by means of a size filter [69]; finally, the seven well-known Hu moments invariant to translations, rotations, and scale changes [70] were computed over each image. As a result of this process, we obtained a five-class dataset problem composed of patterns with seven features.

To reproduce the regular spiking behavior, the parameters for the Izhikevich neuron model were set as *C* = 100, *v*_*r*_ = −60, *v*_*t*_ = −40, *v*_peak_ = 35, *k* = 0.7, *a* = 0.03, *b* = −2, *c* = −50, and *d* = 100. To solve the differential equation, Euler method with *dt* = 1 with a simulation time of *T* = 1000. Finally, the parameters for the artificial bee colony algorithm were set to SN = 40, MAXGEN = 1000, LIM = 100, and the boundaries for the solution to *X*MAX = 10 and *X*MIN = −10.

The classification rate of the model was computed in terms of the number of input patterns correctly classified divided by the total number of tested input patterns. 30 experiments over each dataset were performed to validate the accuracy of the spiking neuron when the ABC algorithm is used as a learning strategy. In order to train the spiking neural model, 80% of the samples (training subset) were randomly selected to do so, the remaining samples (testing subset) were used during the testing phase.

**Figure 3:**
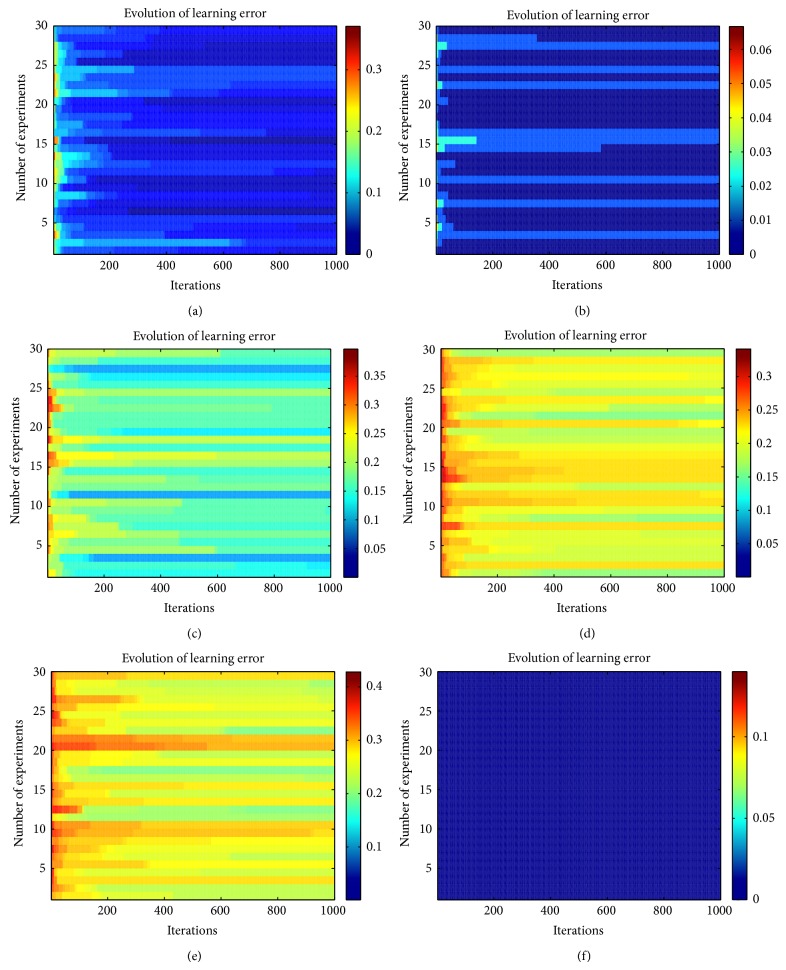
Training error achieved by the spiking model during the learning phase using ABC algorithm. (a) 30 experiments using the wine dataset are shown. (b) 30 experiments using the iris dataset are shown. (c) 30 experiments using the glass dataset are shown. (d) 30 experiments using the diabetes dataset are shown. (e) 30 experiments using the liver dataset are shown. (f) 30 experiments using the object recognition dataset are shown.

At the beginning of the evolutionary learning process, we observed that the learning error rapidly converges to a stable error and after a certain number of generations the learning error changes at a slower rate. Although this behavior was observed in whole experiments, the learning error achieved with the ABC algorithm was not good enough for all the problems. For some problems, the achieved learning error was highly acceptable. In Figure 3 is shown how the learning error evolves through each generation of the ABC algorithm.

After the spiking neuron was trained, we proceed to evaluate the accuracy of the neuron using the remaining 20% samples. From these experiments, we can observe that the performance of the spiking neuron trained with the ABC algorithm was highly acceptable for the six pattern recognition problems. The average percentage of classification computed from all experiments is shown in Table 1. Something that should be remarked is that these methods only used one Izhikevich neuron model. Furthermore, we present the results using the *t*-distribution test to construct the confidence intervals around the mean.

We observed that the classification rate achieved for the iris, wine, and object recognition problems using the spiking neuron trained with the ABC algorithm during the training phase was greater than 96%. However, the classification rate achieved during the testing phase most of the times diminished. Nonetheless, the classification rate was higher than 87%.

On the contrary, for the case of the glass and diabetes problems, we observed that whereas the classification rate achieved using the spiking neuron trained with the ABC algorithm during the training phase was not greater than 84%, the classification rate for the liver problem was not greater than 75%. As a consequence, the accuracy achieved with the spiking neuron drastically diminished during the testing phase. Nonetheless, the results obtained with the spiking neuron trained with the ABC algorithm were acceptable.

**Figure 4:**
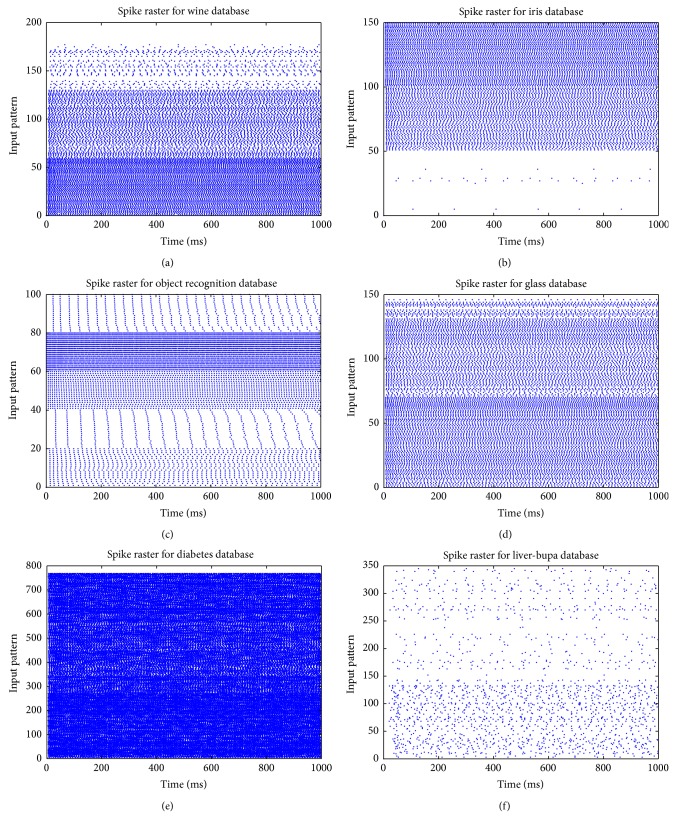
Experimental results obtained with proposed methodology for different datasets. Notice that different firing rates which correspond to different classes can be observed. Each dot represents the time that the neuron generate an spike. (a) Wine dataset. (b) Iris plant dataset. (c) Object recognition dataset. (d) Glass dataset. (e) Diabetes dataset. (f) Liver dataset.

Figures 4(a), 4(b), and 4(c) show the experimental results obtained with the wine, iris plant, and object recognition datasets. Each dot represents the time when the neuron produces a spike. As the reader can observe, the synaptic weights found with the ABC algorithm provoke that the Izhikevich neuron generates almost the same firing rate when it is stimulated with patterns from the same class. Furthermore, the Izhikevich neuron generates firing rates different enough to discriminate patterns that belong to other classes.

On the other hand, Figures 4(d), 4(e), and 4(f) show the experimental results obtained with the glass, diabetes, and liver datasets, respectively. For this pattern recognition problems, the set of synaptic weights found with the ABC algorithm during the training phase were not good enough for the spiking neuron to generate a similar firing rate when it is stimulated with patterns from the same class; as a result, some patterns belonging to the same class were classified in a different class. Although the Izhikevich neuron does not generate firing rates different enough to discriminate among patterns from different classes, the results achieved with the proposed method were quite acceptable.

In addition, in Table 2, we compare the behavior of the methodology using three different swarm intelligence algorithms as a learning strategy: differential evolution (DE) [58, 60], cuckoo search (CS) [61], and particle swarm optimization (PSO) [62].

Furthermore, we perform a comparison against the well-known feedforward neural network (FNN). Two different topologies (1 and 2 hidden layers) and two different training algorithms (descendant gradient and Levenberg-Marquardt) [71] were used to design the FNN. The parameters for training algorithms were set as 0.1 for the learning rate, 2000 epochs for the stop criterion. For each combination (topology-learning algorithm), 30 experiments were done to prove the obtained results statistically. For each experiment, we randomly select the 70% and 10% of the samples for both, training and validation phases. The remaining 20% of the samples were chosen for the testing phase.

The number of neurons that compose the hidden layers depends on the classification problem. The next two topologies were used in combination with each dataset.

1. Wine dataset: 1 HL composes 8 neurons. 2 HL compose 5 and 3 neurons, respectively.
2. Iris plant dataset: 1 HL composes 4 neurons. 2 HL compose 2 and 2 neurons, respectively.
3. Glass dataset: 1 HL composes 8 neurons. 2 HL compose 5 and 3 neurons, respectively.
4. Diabetes dataset: 1 HL composes 5 neurons. 2 HL compose 3 and 2 neurons, respectively.
5. Object recognition dataset: 1 HL composes 6 neurons. 2 HL compose 4 and 2 neurons, respectively.

Table 3 shows the classification efficiencies using feedforward neural networks (FNN). Each column contains the obtained results using a combination of topology and learning algorithms during training and testing phases. Label DG-1 means that the descendant gradient method and one hidden layer (HL) were used to design the FNN, and label LM-2 indicates that Levenberg-Marquardt method and two hidden layers were used to create the FNN, and so on.

The results obtained with the spiking neuron model trained with the ABC algorithm are similar to those obtained in [58, 60–62]. Although with some dataset the ABC algorithm provides better results than DE and PSO algorithm and vice versa, we could not say that one learning strategy is better than the other.

In fact, swarm intelligence algorithms can be considered as a learning strategy to adjust the synaptic weights of a third-generation neural model. After comparing the different algorithms, we note that they present the same behavior and are serious candidates to be considered as a learning strategy. These results suggest that swarm intelligence algorithms combined with spiking neurons can be regarded as an alternative way to perform various pattern recognition tasks. After several experiments, we observed that if we want to improve the efficiency of the methodology, it is not enough to change the learning strategies because they provide similar efficiencies. Nonetheless, the results obtained are highly acceptable.

On the other hand, the reader can observe in Table 2 that the proposed methodology provides better results than traditional FNN composed of several neurons. These results, obtained with the described methodology, show a visible advantage of a spiking model against FNN.

We can also conjecture that if only one neuron is capable of solving pattern recognition problems, perhaps several spiking neurons working together can improve the experimental results obtained in this research.

### 5.2. Application of the Proposed Methodology in a Odor Recognition Problem

In this subsection, we present some preliminary results obtained with the proposed methodology in a problem related to odor recognition. The dataset used was built in Bermak and Martinez [72] where the authors developed an automated gas delivery experimental setup for extracting volatile compounds at given concentrations from liquids, composed of two pumps, two mass flow controllers (MFCs), one bubbler, a gas chamber, and a data acquisition system. This dataset contains patterns obtained from ethanol and butanol vapors injected into the gas chamber at a flow rate determined by the mass flow controllers. The authors also used sensor arrays composed of five commercial TGS Figaro gas sensors (TGS 2600, 2602, 2610, 2611, and 2620). The potential differences across the sensor resistances were measured using a voltage divider with 2.2 k load resistors while keeping the heating voltage constant to 5 V. Finally, the sensors output voltages were sampled at a rate of 10 Hz and quantized with an 11 bit analog to digital converter to build a dataset composed of 124 patterns with 5 features each pattern.

The parameters for the Izhikevich neuron and for the artificial bee colony algorithm and the number of experiments as well as the samples used for training and testing phases were set as equal as in the previous subsection.

Table 4 presents the results achieved with the proposed methodology applied to an odor recognition problem. According to the results presented in Table 4, the reader can observe that the accuracy of the spiking neuron is highly acceptable.

These results obtained with the proposed methodology confirm that spiking neurons can be considered as a pattern recognition technique useful in a wide range of applications, including odor recognition.

### 5.3. Application of the Proposed Methodology in Cancer Classification Based on DNA Microarrays

In this subsection, we present some preliminary results obtained with the proposed methodology for a problem related to cancer classification based on DNA microarrays.

DNA microarrays are a powerful technique in genetic science due to the possibility to analyze the gene expression level of millions of genes at the same time. However, the main problem that arises when DNA microarrays are analyzed with computational intelligent techniques is that the number of genes is too big, and the samples are too few [73].

In that sense, we evaluate the capabilities of a spiking neuron for predict a disease based on the DNA microarrays dataset. It is important to mention that in order to capture the real behavior of the spiking neuron, we do not apply any dimensional reduction technique over the dataset.

The dataset used in this paper was built in [74] where the authors described a generic approach to cancer classification based on gene expression monitoring by DNA microarrays that discover the distinction between acute myeloid leukemia (AML) and acute lymphoblastic leukemia (ALL). The dataset consists of 38 bone marrow samples for training (27 ALL and 11 AML), over 7129 probes from 6817 human genes. Also, 34 samples testing data are provided, with 20 ALL and 14 AML.

The parameters for the Izhikevich neuron and for the artificial bee colony algorithm and the number of experiments were set as equal as in the previous subsection.

Table 5 present the results achieved with the proposed methodology applied to a cancer recognition problem using DNA microarrays. According to the results presented in Table 5, the reader can observe that the accuracy of the spiking neuron is highly acceptable during training phases; however during the testing phase, the accuracy drastically diminished.

These results obtained with the proposed methodology are preliminary and suggest that only one spiking neuron is not enough to solve this problem. Nonetheless, if we combine several spiking neurons to build a network or apply a dimensional reduction stage before training the spiking model, the accuracy could increases. It is important to remark that few highly dimensional samples were used to train the spiking neural model.

## 6. Conclusion

In this paper, we described how the artificial bee colony algorithm can be used as a learning strategy to train a spiking neural model. The results obtained with this approach suggest that ABC algorithm is a useful alternative to adjusting the synaptic weights of the spiking neuron model. Furthermore, we observed that the spiking neuron model provides acceptable results during the pattern recognition task, regardless of the swarm intelligence algorithms used as a learning strategy, the spiking neuron model provides acceptable results during the pattern recognition task.

Although we found that the spiking neuron did not behave as good as we desired with the six datasets, we could say that, in general, the behavior achieved with the spiking neuron provokes that patterns belonging to the same class generate almost the same firing rate in the output of the spiking neuron, and patterns belonging to different classes produce firing rates different enough to discriminate among the different classes. Thanks to the swarm intelligence algorithm used during the evolutionary learning process, the spiking model behaves like we previously described in our starting hypothesis.

Furthermore, a comparison among the ABC, DE, CS, and PSO algorithms was performed. In general, we observe that the accuracy obtained with the spiking neuron model trained with the ABC algorithm is comparable to the accuracy obtained with the methods described in [58, 60–62]. We could not say that one learning strategy is better than the other because, with some datasets, the ABC algorithm provides better results than DE and PSO algorithm and vice versa.

This research provides a clear idea of how powerful a spiking neuron model is in a pattern classification task. Furthermore, the results obtained with the odor recognition and cancer classification problem based on DNA microarrays were highly encouraged.

Nowadays, we are testing different spiking neuron models. Despite the encouraging results achieved with this methodology, we are developing a new method to evolve the synaptic weights at the same time not only for one spiking neuron, but for several spiking neurons as well as to adjust the parameters of the models.

## Figures and Tables

**Pseudocode 1:**
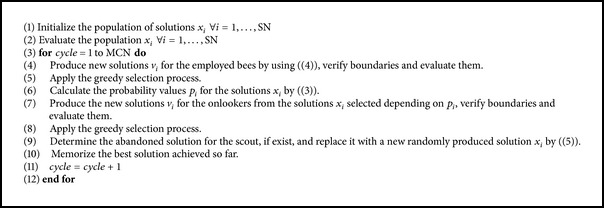


**Table 1:** Statistical results obtained with the proposed methodology.

Dataset	Mean		Confidence intervals	
	Tr. cr.	Te. cr.	TR	TE
Wine	0.963 ± 0.015	0.878 ± 0.038	[0.957–0.968]	[0.864–0.892]
Iris plant	0.996 ± 0.006	0.957 ± 0.025	[0.994–0.999]	[0.948–0.967]
Glass	0.832 ± 0.029	0.703 ± 0.064	[0.822–0.843]	[0.679–0.727]
Diabetes	0.800 ± 0.016	0.743 ± 0.024	[0.794–0.806]	[0.734–0.752]
Liver	0.749 ± 0.024	0.688 ± 0.034	[0.740–0.757]	[0.675–0.700]
Object recognition	1.000 ± 0.000	0.990 ± 0.021	[1.000-1.000]	[0.982–0.998]

Tr. cr = training classification rate, Te. cr. = testing classification rate.

**Table 2:** Average accuracy provided by the methods using different databases.

Dataset	Method using DE		Method using PSO		Method using CS	
	Tr. cr.	Te. cr.	Tr. cr.	Te. cr.	Tr. cr.	Te. cr.
Wine	0.9796	0.8744	0.9782	0.8879	0.9831	0.9078
Iris plant	0.9933	0.9833	0.9933	0.97	0.9942	0.9467
Glass	0.8158	0.7411	0.8178	0.7457	0.8080	0.7646
Diabetes	0.8038	0.7371	0.7990	0.7619	0.8051	0.7477
Liver	0.7620	0.6870	0.7591	0.6754	0.7609	0.6536
Object recognition	1	0.9850	1	0.9950	1	1

Tr. cr = training classification rate, Te. cr. = testing classification rate.

**Table 3:** Average accuracy provided by the FNN using different databases.

	FNN		FNN		FNN		FNN	
Dataset	DG-1		DG-2		LM-1		LM-2	
	Tr. cr.	Te. cr.	Tr. cr.	Te. cr.	Tr. cr.	Te. cr.	Tr. cr.	Te. cr.
Wine	0.9867	0.9800	0.9497	0.9171	0.9986	0.9714	0.9797	0.9629
Iris plant	0.9133	0.8867	0.6625	0.6367	0.9942	0.9767	0.7667	0.7733
Glass	0.6907	0.6738	0.6390	0.6310	0.8453	0.7714	0.7413	0.7119
Diabetes	0.7663	0.7732	0.7153	0.7144	0.7993	0.7320	0.7850	0.7588
Liver	0.5924	0.6145	0.5928	0.5623	0.7243	0.6652	0.7181	0.6812
Object recognition	0.7413	0.6800	0.7413	0.6800	0.7413	0.6800	0.7413	0.6800

Tr. cr = training classification rate, Te. cr. = testing classification rate.

**Table 4:** Statistical results obtained with the proposed methodology.

Dataset	Mean		Confidence intervals	
	Tr. cr.	Te. cr.	TR	TE
Odor	0.952 ± 0.024	0.898 ± 0.064	[0.943–0.961]	[0.874–0.922]

Tr. cr = training classification rate, Te. cr. = testing classification rate.

**Table 5:** Statistical results obtained with the proposed methodology.

Dataset	Mean		Confidence intervals	
	Tr. cr.	Te. cr.	TR	TE
Cancer DNA	0.883 ± 0.024	0.625 ± 0.097	[0.875–0.892]	[0.588–0.661]

Tr. cr = training classification rate, Te. cr. = testing classification rate.
